# Draft genome of spinach and transcriptome diversity of 120 *Spinacia* accessions

**DOI:** 10.1038/ncomms15275

**Published:** 2017-05-24

**Authors:** Chenxi Xu, Chen Jiao, Honghe Sun, Xiaofeng Cai, Xiaoli Wang, Chenhui Ge, Yi Zheng, Wenli Liu, Xuepeng Sun, Yimin Xu, Jie Deng, Zhonghua Zhang, Sanwen Huang, Shaojun Dai, Beiquan Mou, Quanxi Wang, Zhangjun Fei, Quanhua Wang

**Affiliations:** 1 Development and Collaborative Innovation Center of Plant Germplasm Resources, College of Life and Environmental Sciences, Shanghai Normal University, Shanghai 200234, China; 2 Boyce Thompson Institute, Cornell University, Ithaca, New York 14853, USA; 3 Institute of Vegetables and Flowers, Chinese Academy of Agricultural Sciences, Beijing 100081, China; 4 USDA-Agricultural Research Service, Crop Improvement and Protection Research Unit, Salinas, California 93905, USA; 5 USDA-Agricultural Research Service, Robert W. Holley Center for Agriculture and Health, Ithaca, New York 14853, USA

## Abstract

Spinach is an important leafy vegetable enriched with multiple necessary nutrients. Here we report the draft genome sequence of spinach (*Spinacia oleracea*, 2n=12), which contains 25,495 protein-coding genes. The spinach genome is highly repetitive with 74.4% of its content in the form of transposable elements. No recent whole genome duplication events are observed in spinach. Genome syntenic analysis between spinach and sugar beet suggests substantial inter- and intra-chromosome rearrangements during the Caryophyllales genome evolution. Transcriptome sequencing of 120 cultivated and wild spinach accessions reveals more than 420 K variants. Our data suggests that *S. turkestanica* is likely the direct progenitor of cultivated spinach and spinach domestication has a weak bottleneck. We identify 93 domestication sweeps in the spinach genome, some of which are associated with important agronomic traits including bolting, flowering and leaf numbers. This study offers insights into spinach evolution and domestication and provides resources for spinach research and improvement.

## Abstract (web summary)

Spinach is an economically important vegetable crop but previous genomic resources were of limited use for comparative and functional analyses. Here, Xu *et al*. present a high quality draft spinach genome and transcriptome data for multiple *Spinacia* accessions providing insight into Caryophyllales genome evolution.

Spinach (*Spinacia oleracea* L., 2n=2 × =12) is an important and nutritious green leafy vegetable and a rich source of carotenoids, folate, vitamin C, calcium and iron. It is commonly used as a salad, a cooked vegetable or as an ingredient in fresh or cooked meat and vegetable dishes^1^. Spinach is increasing in popularity and is cultivated in more than 60 countries with production increased nearly tenfold in the past 40 years (annual production of ∼2.5 million tonnes in 1974 and ∼23 million in 2013; http://faostat3.fao.org/).

Spinach is an annual or biennial plant, and is commonly considered as dioecious or occasionally monoecious^2^. It is native to central Asia and thought to have originated in Persia (Iran)^1^. The genus *Spinacia* contains two wild species, *S. turkestanica* Ilj. and *S. tetrandra* Stev., which are considered the probable ancestors of cultivated spinach^3^. Spinach has a relatively recent domestication history^4^. In addition to common horticultural traits (for example, high yields, fast growth, slow bolting and leaf type), disease resistance breeding has been the major focus of spinach improvement programmes, especially resistance to the downy mildew pathogen, *Peronospora farinosa* f.sp. s*pinaciae* (*Pfs*)^1^,^4^. Several studies have also demonstrated that wide variations in important traits including carotenoid and folate concentrations^1^,^5^ and oxalate and nitrate contents^6^,^7^ exist in spinach germplasm, providing new breeding targets for spinach improvement. Nevertheless, genetic diversity among spinach germplasm remains largely unexplored at the molecular level with only limited studies spanning useful but relatively small collections of spinach accessions^8^,^9^,^10^,^11^. Furthermore, due to its minor crop status, few genomic resources have been developed, hindering the understanding and utilization of the extant germplasm resources.

Spinach is a member of the Amaranthaceae family, which is composed of about 180 genera and 2,500 species including important crops such as beets and quinoa, and represents the most species-rich lineage within Caryophyllales, the basal order of core eudicots^12^. Previously a draft spinach genome assembly was generated, which was mainly used to provide evidence to support the separation of Caryophyllales from rosids and asterids^12^. However, the assembly (498 Mb) represents only about half of the genome with an estimated size of 989 Mb (ref. 13) and contains many short assembled fragments (N50=19 kb), thus limiting its utility as a reference for comparative, evolutionary and functional genomic studies.

Here we report a high-quality genome assembly of a Chinese spinach cultivar, Sp75, in addition to transcriptome sequences of 120 cultivated and wild spinach accessions. These sequences provide insights into the structure and evolution of the spinach genome, the phylogeny and genetic diversity of spinach populations, genomic signatures underlying spinach domestication, and the underlying molecular basis of specific agronomically important traits. The spinach genome sequence represents a solid foundation for comparative genomic studies in Caryophyllales and eudicots, and together with the transcriptome variation data, provide valuable resources for facilitating spinach research and improvement.

## Results

### Spinach genome sequencing and assembly

**Figure 1:**
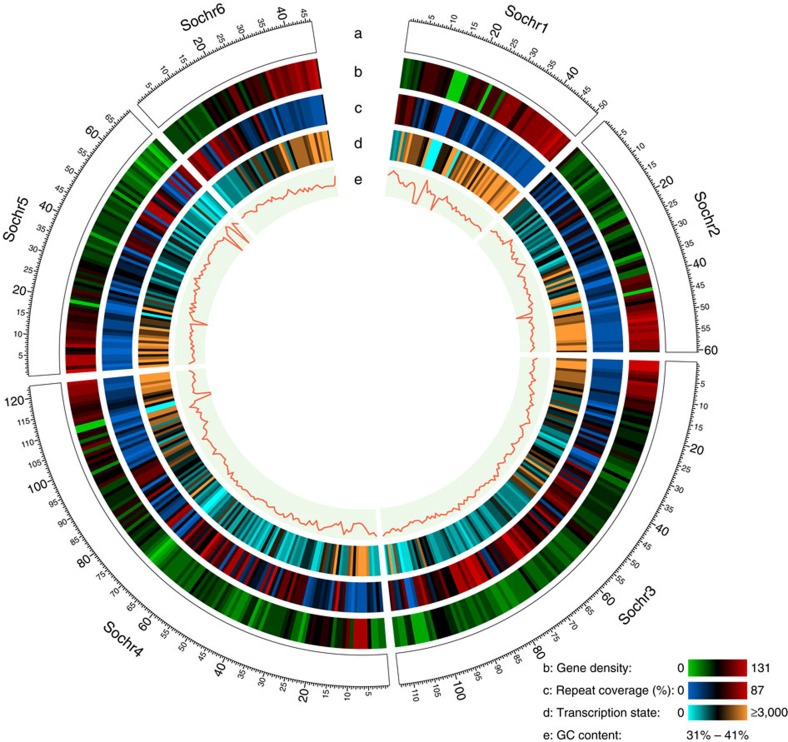
Spinach genome landscape. (**a**) Ideogram of the six spinach pseudochromosomes (in Mb scale). (**b**) Gene density represented as number of genes per Mb. (**c**) Percentage of coverage of repeat sequences per Mb. (**d**) Transcription state. The transcription level was estimated by read counts per million mapped reads in 1-Mb windows. (**e**) GC content in 1-Mb windows. The six spinach pseudo-chromosomes represented 47% of the genome assembly. This figure was generated using Circos (http://circos.ca/).

An inbred spinach line, Sp75, was selected for reference genome sequencing using the whole genome shotgun approach. Illumina paired-end libraries with insert sizes of 150 bp, 200 bp, 300 bp, 500 bp and 1 kb, and mate-pair libraries with insert sizes of 3 kb, 10 kb and 15 kb were constructed and sequenced, which generated a total of 169 Gb high-quality cleaned sequences (Supplementary Table 1), representing approximately 168-fold coverage of the spinach genome that has an estimated size of 1,009 Mb based on k-mer analysis of the Illumina sequences (Supplementary Fig. 1) and 989 Mb based on flow cytometry^13^. *De novo* assembly of the Illumina sequences resulted in a draft genome of ∼870 Mb with an N50 scaffold length of ∼319.5 kb and the longest scaffold of 3.3 Mb (Table 1). To further improve the assembly, we constructed spinach genome maps using the BioNano Irys system^14^. A total of 808,135 molecules (>150 kb) with a total length of 214.9 Gb were collected, representing ∼213 × coverage of the spinach genome. *De novo* assembly of these molecules resulted in a total of 898 genome consensus maps with a total length of 1,087.6 Mb and individual map lengths ranging from 49.8 kb to 8.9 Mb. Reconstruction of the genome assembly using BioNano consensus maps resulted in super-scaffolds with a total length of ∼996 Mb, N50 of 919,290 bp, and the longest of 9.3 Mb (Supplementary Fig. 2). Furthermore, using a genetic map we constructed, we were able to anchor a total of 439 scaffolds covering 463.4 Mb (47%) of the assembled genome to the six linkage groups (Fig. 1, Supplementary Note 1 and Supplementary Figs 3–5).

The quality of the spinach genome assembly was first assessed using BUSCO^15^. The analysis revealed that 97.1% of the core eukaryotic genes were detected in the spinach genome, and 95.7% of them were completely covered. Furthermore, RNA-Seq data sets generated from the 107 *S. oleracea* samples were used to evaluate the quality of the assembled spinach genome. Overall, ∼95% of the RNA-Seq reads could be mapped to the assembled spinach genome (Supplementary Data 1). In summary, the high coverage of the core eukaryotic genes and the high mapping rate of RNA-Seq reads indicated the high quality of the assembled genome.

### Transposable elements and gene models

The assembled spinach genome contains a total of ∼618 Mb (74.4%) of repeat sequences, higher than that in most eudicot genomes reported to date. Class I transposons are the major source of repeat sequences, covering 59.5% of the genome. The predominant subgroup in the class I transposon family is the long terminal repeat (LTR) retrotransposons, which accounts for 56.6% of the genome assembly (Supplementary Table 2). In contrast, class II or DNA transposons are much less prevalent (‘cut-and-paste' transposons, 6.0%; miniature inverted repeat transposable elements (MITEs), 2.9%; and helitron, 0.1%). Although LTR retrotransposons can be eliminated efficiently from the genome, upon proliferation or transposition burst, they can substantially increase the genome size in a short evolutionary time^16^. Sugar beet, another Caryophyllales species closely related to spinach, has a smaller genome size (760 Mb) and a lower content of repetitive sequence (42%) in its assembled genome^12^. We identified 976 and 498 full-length LTR retrotransposons in our spinach assembly and the sugar beet genome, respectively. We found that there was a more recent burst of LTR retrotransposons in spinach, which was estimated to occur at ∼1.5 million years ago (Mya), compared to that in sugar beet occurring at ∼2.7 Mya (Supplementary Fig. 6). This may partially explain the larger genome size and higher repeat content in spinach as compared to sugar beet. Transposon amplification has also been reported to contribute to the differences in genome sizes of other closely related species, such as melon and cucumber^17^.

A total of 25,495 protein-coding genes were predicted from the spinach genome, which is comparable to the number of genes predicted in sugar beet (26,923; RefBeet-1.2) (ref. 18). Among the predicted spinach protein-coding genes, 22,860 (89.7%) were supported by our RNA-Seq data. The coding sequences in spinach have an average length of 1,157 bp and the predicted genes have an average of 5.3 exons; both are similar to sugar beet (Supplementary Table 3). A total of 22,103 (86.7%) spinach proteins have homologues in at least one protein database including nr, TrEMBL, Swiss-Prot and TAIR10, 19,620 (77.0%) and 18,725 (73.5%) contain InterPro and Pfam domains, respectively, and 17,744 (69.6%) can be assigned with Gene Ontology (GO) terms (Supplementary Table 4). Furthermore, a total of 1,202 transcription factors were identified in the spinach genome, slightly more than sugar beet but less than non-Caryophyllales plant species (Supplementary Table 5). Moreover, the spinach genome encodes 892 protein kinases and 68% of them belong to the receptor-like kinase family. One receptor-like kinase group, the WAK family, was found to be expanded in basal eudicots spinach and sugar beet and monocot rice as compared to other analysed eudicot species (Supplementary Table 6).

### Comparative genomics

**Figure 2:**
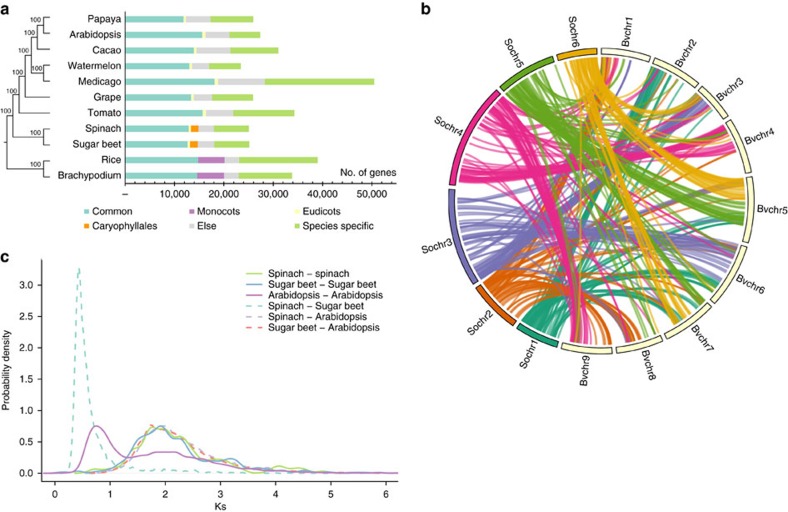
Comparative genomic analysis among spinach and other plant species. (**a**) Phylogenetic relationship and gene clusters of 11 plant species. A maximum parsimony (MP) species tree (left) was constructed using protein sequences of the 2,047 single-copy genes. Bars (right) represent the number of genes in different categories for each species. Common: genes that are found in at least 10 of the 11 species. Monocots: genes that are only found in the two monocots, rice and *Brachypodium*; Eudicots: genes that are found in at least eight of nine eudicots but not in the two monocots; Caryophyllales: genes that are only found in spinach and sugar beet; Species-specific: genes with no homologues in other species. (**b**) Syntenic relationships between spinach and sugar beet genomes. (**c**) *Ks* distribution of homologous gene pairs in spinach, sugar beet and *Arabidopsis.* The probability density of *Ks* was estimated using the ‘density' function in R.

Comparison of gene content between spinach and other ten representative plant species, including sugar beet, tomato, grape, *Medicago*, watermelon, cacao, *Arabidopsis*, papaya, *Brachypodium* and rice (Supplementary Data 2), indicated that 17,968 (71.7%) spinach genes were present with homologues in at least one of the other ten species. A total of 1,520 (6.1%) spinach genes were Caryophyllales specific (unique to spinach and sugar beet), and 7,097 (28.3%) were spinach specific (having no homologues in the other ten species). A phylogenetic tree constructed using single-copy genes from all 11 species confirmed that spinach is a sister taxon of sugar beet, and supported that these two species constituted the clade which is the most basal in core eudicots^12^ (Fig. 2a). Despite that the spinach pseudo-chromosomes only represented 47% of the genome assembly, our analysis strongly indicated that the genomes of spinach and sugar beet shared many syntenic regions, whose pattern clearly suggested that substantial inter- and intra-chromosome rearrangements have occurred during the Caryophyllales genome evolution (Fig. 2b).

Whole genome duplication is prevalent in flowering plants and is an important driving force for genome evolution and gene neofunctionalization^19^. We identified homologous pairs in the syntenic genomic regions within and between spinach, sugar beet and Arabidopsis. The distribution of synonymous substitution rate (*Ks*) of the homologous pairs indicated that as expected an ancient whole genome triplication (the *γ* event), which is shared by core eudicots, had occurred in the evolutionary history of the spinach genome. However, no recent whole genome duplication event was found in spinach, nor in sugar beet^12^ (Fig. 2c). In addition, the divergence of spinach and sugar beet occurred approximately 38.4 Mya (95% confidence interval: 38.1–38.7 Mya). The common ancestor of spinach and sugar beet diverged from Arabidopsis soon after the ancient triplication event (*γ*) (Fig. 2c).

### Disease resistance genes

Currently, resistance against downy mildew is the major focus of spinach breeding^1^. Recognition of pathogen effector is mainly mediated by plant disease resistance (*R*) genes, which encode nucleotide-binding site leucine-rich repeat (NBS-LRR) proteins^20^. We identified 139 NBS-LRR genes in the spinach genome and classified them into six categories based on their Toll/Interleukin-1 receptor domains, coiled coil (CC) motifs and LRRs (Supplementary Data 3). The number of NBS-LRR genes in spinach is lower than that in most other plant species (Supplementary Fig. 7a). The two most abundant groups of NBS-LRR genes in spinach are CNL (coiled coil, NBS and LRR) and NL (NBS and LRR), representing 40 and 36% of the NBS-LRR genes, respectively. Comparison of NBS-LRR genes between spinach and other five representative plants (sugar beet, tomato, Arabidopsis, rice and moss) in different orders confirmed that NBS-LRR genes are generally expanded in higher plants, but to different extents and in different subclasses (Supplementary Fig. 7a). Interestingly, same as reported in Dohm *et al*.^12^, we also found that the two Caryophyllales, spinach and sugar beet, harboured only one TNL gene, while the TNL family is highly expanded in other eudicots, for example, 19 members in tomato and 78 in Arabidopsis (Supplementary Fig. 7a–b).

It was reported that a sequence characterized amplified region (SCAR) marker, DM-1, was tightly linked to the *Pfs-1* locus and could distinguish spinach genotypes that were homozygous resistant, heterozygous resistant or homozygous susceptible to race 6 of *Pfs*^21^. We mapped the DM-1 marker to the spinach genome and identified five NBS-LRR genes close to the marker, of which two pairs were tandemly duplicated (Supplementary Fig. 7c). As such, these are potentially candidate genes for spinach resistance to race 6 of *Pfs*.

### Transcriptome diversity and population analysis

**Figure 3:**
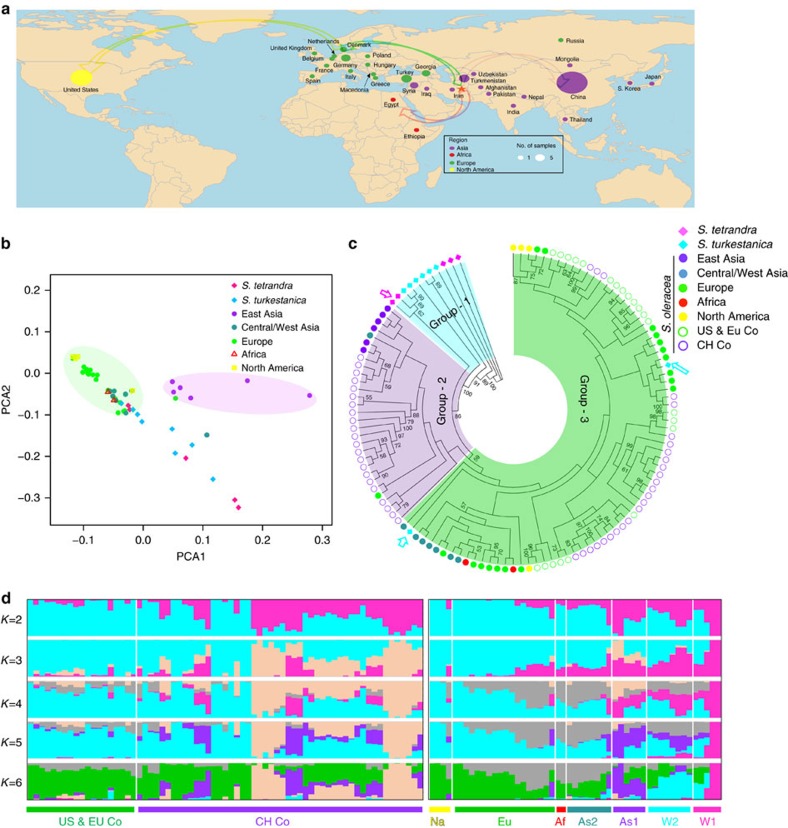
Geographic distribution and population structure of the 120 spinach accessions. (**a**) Geographic information of the 120 accessions. The number of samples is represented by the dot size on the world map. The red star indicates the suggested origin location of spinach, and the arrows suggest the domestication history of spinach. Commercial cultivars provided by Chinese seed companies are plotted in China and commercial cultivars provided by American/European companies are plotted in United States. (**b**) PCA plot of non-commercial spinach accessions. (**c**) Phylogenetic tree of all spinach accessions inferred from transcriptome SNPs, with *S. tetrandra* Sp42 and Sp43 as the outgroup. The pink arrow indicates the two *S. tetrandra* (Sp39 and Sp40) that were grouped to *S. turkestanica* and the blue arrows indicate the two *S. turkestanica* (Sp47 and Sp48) that were clustered with cultivars. (**d**) Model-based clustering analysis of the 51 non-commercial (right) and 69 commercial (left) spinach accessions, given different number of groups (*K*=2 to 6). The *y* axis quantifies subgroup membership, and the *x* axis shows different accessions. W1: *S. tetrandra*; W2: *S. turkestanica*; As1: East Asia; As2: Central/West Asia; Eu: Europe; Af: Africa; Na: North America; CH Co: companies in China; US & EU Co: companies in United States and Europe.

Unlike other major crops, spinach has a much smaller collection of genetic stocks^1^, especially for the wild species, with only a small number of accessions available in public gene banks^3^. To assess genetic diversity and infer population structure of spinach germplasm, we performed transcriptome sequencing of 120 spinach accessions including 107 cultivated *S. oleracea* and 13 wild accessions (5 *S. tetrandra* and 8 *S. turkestanica*). The cultivated *S. oleracea* accessions originated from diverse sources including Asia (14), Europe (18), Africa (2), North America (4), and 49 more provided by seed companies in China and 20 by companies in Europe or America (Fig. 3a and Supplementary Data 1). We generated a total of approximately one billion RNA-Seq reads (∼100 Gb in total) and more than 90% of the accessions had more than 5 million reads (Supplementary Data 1). Mapping of this large-scale RNA-Seq data set to the spinach reference genome identified a total of 420,545 single nucleotide polymorphisms (SNPs) and 12,618 small insertions and deletions (indels) (Supplementary Table 7). A previous study by Fujito *et al*.^22^ indicated that two accessions, Sp39 (Ames 23664) and Sp40 (PI 608712), were wrongly classified as *S. tetrandra*. Our phylogenetic analysis (see below), combined with findings from Fujito *et al*.^22^, suggested that Sp39 and Sp40 might belong to *S. turkestanica*. Nonetheless, we excluded these two accessions in counting population SNPs and genetic diversities. *S. oleracea*, *S. tetrandra* and *S. turkestanica* possessed ∼192.5 k, ∼117.3 k and ∼52.0 k SNPs, respectively (Supplementary Table 7). As expected, most SNPs (65%) were located in the coding regions, among which 43% were nonsynonymous substitutions (Supplementary Table 8), and the remaining SNPs (35%) were located in untranslated regions, introns of alternatively spliced transcripts and intergenic regions corresponding to non-coding RNAs. Moreover, 6,080 SNPs were located at either start or stop codons, or splice site acceptors or donors, impacting 4,415 genes. In addition, we also identified 1,358 genes that harbour small indels causing frame shifts or indels of amino acid sequences.

We then estimated the nucleotide diversity (*π*) in transcriptomes of spinach populations. The average values of *π* for *S. oleracea, S. turkestanica* and *S. tetrandra* were 0.67 × 10^−3^, 0.83 × 10^−3^ and 6.40 × 10^−3^, respectively (Supplementary Table 9). The nucleotide diversity in the group of wild *S. tetrandra* was almost ten times of that in the group of *S. oleracea* or *S. turkestanica*, indicating a highly diverse gene pool in *S. tetrandra* that contains valuable genetic diversity for spinach improvement.

Principal component analysis of spinach accessions (excluding those from commercial companies due to their ambiguous geographic background) showed that those from East Asia and those from other regions were roughly divided into two groups, and *S. turkestanica* accessions were generally more closely related to the cultivated ones than *S. tetrandra* (Fig. 3b). The observation was further supported by the phylogenetic analysis, where spinach accessions were clustered into three major groups (Fig. 3c). The first group consisted of *S. turkestanica* and *S. tetrandra* accessions, the second group contained cultivars from East Asia, Chinese commercial varieties and two cultivars from Pakistan and Russia, and the third group included cultivars from Central/West Asia, Europe, North America and Africa, as well as the remaining commercial cultivars (Fig. 3c). These results suggest that cultivated spinach was likely domesticated directly from the wild species *S. turkestanica*. The East Asian cultivars and cultivars from Central/West Asia had a common ancestor but evolved separately. Moreover, the phylogenetic tree indicates that the European spinaches were derived from the varieties from Central/West Asia while the North American spinaches were descendants of European ones (Fig. 3c). We found two *S. turkestanica* samples (Sp47 and Sp48) that were clustered with varieties from Europe, America, Africa and West Asia (Fig. 3c). Sp47 was re-classified as a *S. hybr* recently in the National Plant Germplasm System (http://www.ars-grin.gov/npgs), and Sp48 showed mixed traits of wild and cultivated spinaches, indicating it could also be a hybrid between a wild and a cultivated accession.

We further used the model-based program STRUCTURE^23^ to estimate individual ancestry and admixture proportions using the SNP data. Our analysis indicated that *K*=2 ancestral types best explained the current population structure (Supplementary Fig. 8). Compared to other annual crops (for example, tomato, watermelon and cucumber), wild and cultivated spinach populations have more complex subpopulation structures, which might be partially due to the outcrossing nature of spinach. However, the cultivated spinaches, especially those from Europe and North America, were genetically more homogeneous than the wild groups (Fig. 3d). The ancestor–descendant relationships among the Central/West Asian, European/African and North American groups, as well as the reduced structure complexity during the distribution of spinach from its origin to the other parts of the world, are in agreement with the domestication history of spinach, which was native to Central Asia in Iran (Persia), introduced to North Africa and Europe around 1,100 A.D. by Arabs, and then brought to North America by the early colonists^4^ (Fig. 3a).

We investigated the linkage disequilibrium (LD) decay patterns of spinach, and found that the decay of LD with the physical distance between SNPs occurred at 5 kb in cultivated *S. oleracea* and 4 kb in its progenitor (*S. turkestanica*) accessions (*r*^2^=0.2) (Supplementary Fig. 9). The relatively rapid LD decay in cultivated spinach indicates a weak bottleneck during its domestication.

### Domestication and population differentiation of spinach

**Figure 4:**
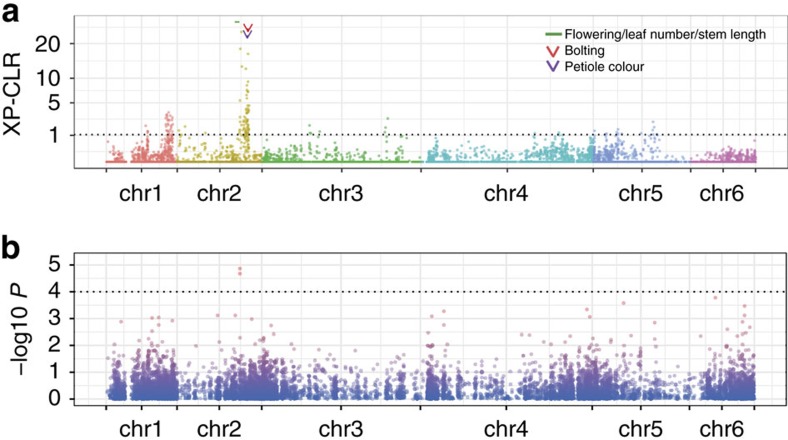
Genome-wide scan of selective sweeps and GWAS of bolting. (**a**) Distribution of XP-CLR scores across the spinach genomes. The black horizontal dashed line refers to the top 1% threshold. Arrows and the short interval indicate positions of known SNP markers and QTL, respectively, for different traits. (**b**) Manhattan plot of the GWAS for spinach bolting trait. The significance threshold (1 × 10^−4^) is indicated by the black horizontal dashed line.

According to multiple lines of evidence, domestication of spinach started at ∼2,000 years ago^24^. Unfortunately, no direct historical record or research of early domesticated traits is currently available. Spinach is thought to have originated in Persia and then introduced and cultivated worldwide. During the spread of spinach to East Asia, Europe and North America, local gardeners and breeders started to improve traits such as yield, leaf quality and bolting resistance^25^. These improvements may have arisen independently in various spinach-growing areas^4^. Domestication often imposes a significant reduction of genetic diversity and enriches genetic properties favoured by humans^26^. However, in spinach we found a very small difference of genome-wide genetic diversity between *S. oleracea* (*π*=0.67 × 10^−3^) and its progenitor *S. turkestanica* (*π*=0.83 × 10^−3^), further supporting a weak bottleneck during its domestication. Nonetheless using the transcriptome variant data, we were able to identify potential selective signals in the spinach genome by scanning extreme allele frequency differentiation over extended linked regions using an across-population likelihood method implemented in XP-CLR^27^. A total of 93 regions (∼2.3 Mb in total), ranging from 10 kb to 150 kb in length (24 kb on average), were identified as candidate domestication sweeps that involved 261 (1.0%) protein-coding genes (Fig. 4a and Supplementary Data 4). Genes in the selected regions were significantly enriched with those having the fucosidase activity (GO:0015928, adjusted *P*=0.00142, hypergeometric distribution test), receptor signalling protein serine/threonine kinase activity (GO:0004702, adjusted *P*=0.00919), or signal transducer activity (GO:0004871, adjusted *P*=0.01593), implying their potentially important roles in spinach domestication.

A large number of sweeps with strong selection signals were located at a region from 44.7 to 50.5 Mb of chromosome 2. Interestingly, this region contains a number of known markers and quantitative trait loci (QTLs) associated with several potential domestication traits in spinach such as bolting (Fig. 4a and Supplementary Data 5). We further performed a genome-wide association study for the bolting trait in 59 spinach accessions. The strongest association signals also resided in this region and overlapped with the selective sweep with the second highest XP-CLR score (Fig. 4b). In addition, one flowering QTL and one gene (*Spo00403*) showing high homology to the Arabidopsis *AGAMOUS-like 20*, which has been reported to control flowering time^28^, were also covered by the sweeps in this region (Fig. 4a and Supplementary Data 4 and 5). Furthermore, QTLs for leaf number and stem length, and an SNP marker associated with petiole colour, were also found in the sweeps (Fig. 4a and Supplementary Data 5). Taken together, our data provide insights into the genomic basis of the potential domestication traits in spinach.

We next investigated the population differentiation using the *F*_ST_ statistic for each sliding-window between *S. oleracea* and *S. turkestanica*. The windows with top 1% *F*_ST_ values (>0.3907) were merged into 103 genomic regions, covering 222 genes (Supplementary Data 6). QTLs for spinach flowering, and SNP markers associated with bolting, petiole colour and erectness traits were covered by these highly divergent regions (Supplementary Fig. 10).

## Discussion

We report a high-quality draft genome sequence of spinach, which was assembled using high coverage of Illumina sequences and BioNano genome maps. The spinach genome sequence provides an important resource for future comparative genomic and evolutionary studies, especially in the order Caryophyllales that constitutes the basal clade in core eudicots^12^. The spinach genome is among the most highly repetitive plant genomes reported to date, and much larger than the genome of its closely related species sugar beet, likely due to a recent burst of LTR retrotransposons after speciation. However, the two genomes show high collinearity and no recent whole genome duplications have occurred during the evolution of both genomes. Furthermore, our analysis indicates substantial chromosome fissions, fusions and rearrangements during the evolution of the Caryophyllales genomes.

Currently little information is available on spinach evolution and domestication. Through the analysis of transcriptome variants from a large collection of cultivated and wild spinach accessions, we infer that wild *S. turkestanica* could be the direct progenitor of cultivated *S. oleracea* and spinach domestication has a very weak bottleneck. A genome-wide scan for domestication signatures revealed 93 selective sweeps in the spinach genome that contain a number of QTLs and markers that are known to be associated with potential domestication traits in spinach such as bolting, flowering, leaf number and stem length. Furthermore, a set of highly divergent genomic regions were identified between wild and cultivated spinach species, which also contain QTLs and markers associated with important spinach traits, providing important information for facilitating spinach breeding.

Our comprehensive genome and transcriptome analyses provide novel insights into spinach genome architecture and evolution, disease-resistance and horticultural traits, and spinach genetic diversity, evolution and domestication. Furthermore, the genome sequence and transcriptome variant data provide valuable resources for facilitating spinach research and improvement.

## Methods

### Plant materials and library preparation and sequencing

A sibling inbred line, Sp75, was grown under standard greenhouse conditions with a 16-h light (27 °C) and 8-h dark (19 °C) cycle. Young leaves from 20-days-old plants were collected, immediately frozen in liquid nitrogen and stored at −80 °C. DNA was extracted using the QIAGEN DNeasy Plant Mini Kit following the manufacturer's instructions (QIAGEN, Valencia, CA, USA). DNA quality was evaluate via agarose gel electrophoresis and its quantity was determined on a NanoDrop (Thermo Fisher Scientific, Waltham, MA, USA). Paired-end libraries with insert sizes of 150 bp, 200 bp, 300 bp, 500 bp and 1 kb and mate-pair libraries with insert sizes of 3, 10 and 15 kb were constructed using the Genomic DNA Sample Prep kit and the Nextera Mate Pair Sample Preparation kit (Illumina, San Diego, CA, USA), respectively, following the manufacturer's instructions, and sequenced on an Illumina HiSeq 2500 platform with paired-end mode.

### Genome assembly and quality evaluation

Raw Illumina reads were processed to collapse duplicated read pairs into unique read pairs. Duplicated read pairs were defined as those having identical bases at positions of 14 to 90 in both left and right reads. The resulting reads were further processed to remove adaptor and low quality sequences using Trimmomatic^29^ (v0.33; parameters ‘SLIDINGWINDOW:4:20 LEADING:3 TRAILING:3 MINLEN:40'). Furthermore, sequences of the junction adaptor used in the mate-pair library construction were identified and removed together with the trailing bases using the ShortRead package^30^. Reads shorter than 40 bp were discarded. Finally, sequencing errors in paired-end reads were corrected with QuorUM^31^ (parameter ‘--kmer-len 24').

The high-quality cleaned paired-end reads were first assembled into contigs using Platanus^32^ (v1.2.1; parameters ‘-k 32 -s 10 -c 2 -a 10.0 -u 0.1 -d 0.5'). The resulting contigs were first processed by a module ‘Prepare' released with SOAPdenovo2 (v2.04; ref. 33) with ‘-D' option, then connected to scaffolds with SOAPdenovo2 using all paired-end and mate-paired reads. To further improve the assembly, we generated three additional assemblies using different combinations of contig assembly and scaffolding programs: (1) contig assembly with Platanus and scaffolding with SSPACE^34^ (parameters ‘-x 0 -m 32 -o 20 -t 0 -k 5 -a 0.70 -n 15'); (2) both contig assembly and scaffolding with Platanus; and (3) both contig assembly and scaffolding with SOAPdenovo2 (parameters ‘-K 60 -m 127 -M 2 -d 1 -R'). Reads from the 15-kb insert mate-pair library were then used to break any potential scaffolding errors in these four assemblies with REAPR^35^ (v1.0.17; parameter ‘-score f=10'). Using the first assembly (Platanus+SOAPdenovo2) as the backbone, the other three assemblies were integrated. First, the three assemblies were aligned to the first assembly using LAST^36^ (v604; parameters ‘-r 1 -q 3 -a 7 -b 1 –e 80'), and based on the alignments local collinear blocks were determined by Mugsy^37^ (v1.2.3; parameters ‘-d 1000 -c 30 -duplications 0'). Scaffolds from the first assembly were then connected if they were aligned adjacently in the same scaffold in the other assemblies without any conflicts. Gaps in the final consensus scaffolds were filled using gapcloser (v1.12; parameter ‘-p 25') in the SOAPdenovo2 package^33^ with paired-end reads. The assembly was further polished to correct base errors using Pilon^38^ (v1.8) with paired-end reads.

### BioNano genome map construction and integration

High molecular weight DNA was extracted from spinach Sp75 seedlings by Amplicon Express (Pullman, WA). Labeling of high molecular weight DNA molecules and BioNano map data collection were performed according to Pendleton *et al*.^39^ using the IrysPrep Reagent Kit (BioNano Genomics, San Diego, CA, USA). Single molecule maps longer than 150 kb were used for *de novo* assembly using IrysView (BioNano Genomics) with *p* value threshold set to 1 × 10^−8^. The resulting consensus maps (CMAPs) were then used to join the assembled spinach scaffolds to super-scaffolds using the ‘Sewing machine pipeline'^40^ with parameters of ‘--f_con 13 --f_algn 30 --s_con 8 --s_algn 90 -T 1e-8'. The gap lengths between assembled genome scaffolds were estimated by BioNano CMAPs.

### Annotation of transposable elements

A *de novo* LTR retrotransposon library and a MITE library were constructed by screening the assembled spinach genome using LTRharvest^41^ (v1.5.7; parameters ‘-minlenltr 100 -maxlenltr 6000 -mindistltr 1500 -maxdistltr 25000 -mintsd 5 -maxtsd 5 -motif tgca -vic 10') and MITE-Hunter^42^ (v11-2011; parameters ‘-n 20 -c 50'), respectively. The spinach genome was then masked using RepeatMasker (v4.0.5; http://www.repeatmasker.org/) with the LTR retrotransposon and MITE libraries, and the unmasked sequences were further searched for repeat elements using RepeatModeler (v1.0.8; http://www.repeatmasker.org/RepeatModeler.html). All the repetitive sequences generated above were combined into a single repeat library, and then compared against the Swiss-Prot database (http://www.uniprot.org/) using BLAST with an e-value cutoff of 0.01. Sequences matching any non-TE proteins in the database were removed from the repeat library. TEs in the library were classified using REPCLASS^43^ (v1.0.1). The repeat library was then used to identify TEs in the assembled spinach genome with RepeatMasker (parameters ‘-s -x -nolow -norna -no_is -a').

### LTR retrotransposon insertion time analysis

We used LTRharvest^41^ (v1.5.7) to *de novo* detect full length LTR retrotransposons in both sugar beet and spinach genomes with parameters of ‘-motif tgca -motifmis 1 -minlenltr 100 -maxlenltr 3000 -mintsd 4 -maxtsd 20'. Domains located in internal regions of LTR retrotransposons including reverse transcriptase, protease, ribonuclease H and integrase were identified using LTRdigest^44^. Only LTR retrotransposons containing all four domains were used for the insertion time analysis. The two ends of these LTR retrotransposons were aligned with MUSCLE^45^ (v3.8.31) and the distance was calculated using the distmat program in the EMBOSS package^46^ (v6.5.7) with parameter of ‘-nucmethod 2'. The insertion time (*T*) of an LTR retrotransposon was calculated using the formula *T*=*K*/2*r*, where *K* is the distance and *r* is the rate of nucleotide substitution, which was set to 7.0 × 10^−9^ substitutions per site per year according to Ossowski *et al*.^47^.

### Protein-coding gene prediction and functional annotation

The repeat-masked spinach genome was used for gene prediction using MAKER^48^ (v2.31.6), which combines evidence from *ab initio*, transcript mapping and protein homology-based predictions to define the confident gene models. SNAP^49^ (v2009-02-03) and AUGUSTUS^50^ (v3.0.2) were used for *ab initio* gene predictions. For transcript mapping, RNA-Seq data were aligned to the spinach genome using Tophat^51^ (v2.0.13; parameters ‘--read-mismatches 1 --splice-mismatches 0 --min-intron-length 30') and then assembled using Cufflinks^52^ (v2.2.1; parameters ‘--no-effective-length-correction --min-intron-length 30 --min-frags-per-transfrag 5'). For homologous protein mapping, protein sequences from sugar beet, Arabidopsis, potato, tomato and Swiss-Prot were aligned to the genome using Spaln^53^ (v2.1.4; parameters ‘-Xk11 -H35'). For predicted genes without a transcript or homologous protein support, they were compared against the Pfam database (http://pfam.xfam.org) and those that did not contain any Pfam domains were discarded. Furthermore, genes highly homologous to transposable elements were also removed. Predicted genes with expression levels of at least one RPKM (reads per kilobase of exon model per million mapped reads) in at least one of the 120 spinach accessions were considered as supported by the RNA-Seq data.

To annotate the spinach predicted genes, their protein sequences were compared to GenBank nr, the Arabidopsis protein and UniProt (Swiss-Prot and TrEMBL; http://www.uniprot.org/) databases using BLAST (parameter ‘-evalue 1e-4'), as well as the InterPro database using InterProScan^54^ (v5.10–50.0). GO annotations were obtained using Blast2GO (ref. 55) (version 2.5.0) based on the BLAST results against the GenBank nr database and results from the InterProScan analysis. Functional descriptions were assigned to spinach genes using AHRD (v3.3; https://github.com/groupschoof/AHRD). Enzyme-encoding genes were extracted based on the AHRD result and Enzyme Commission (EC) information from the Blast2GO analysis. Transcription factors and protein kinases were identified and classified into different families using the iTAK pipeline^56^ (v1.6).

### Comparative genomic analyses

Orthologous groups were constructed using OrthoMCL^57^ (v1.4; inflation parameter: 1.5) with an all -versus-all BLASTP comparison (parameters ‘-evalue 1e-5 -max_target_seqs 1000') of protein sequences from spinach and ten other plant species, including *Brachypodium distachyon*, rice, sugar beet, tomato, grape, *Medicago truncatula*, watermelon, cacao, *Arabidopsis thaliana* and papaya (Supplementary Data 2). Protein sequences of single-gene families were aligned by MUSCLE^45^ (v3.8.31). The alignments were trimmed by the trimAL program^58^ (v1.2rev59) with the parameter ‘gt' set to 0.9. The concatenated protein alignments were used to construct a phylogenetic tree using the maximum parsimony method implemented in MEGA6 (ref. 59) with 1,000 bootstrap replications. Syntenies within and between spinach, sugar beet and Arabidopsis genomes were detected using MCScanX^60^ (parameters ‘-s 5 -e 5'). *Ks* values of homologous gene pairs in the syntenic regions were calculated and the speciation time base on *Ks* values was derived using the equation *T*=*Ks*/2*r* with *r*=7.0 × 10^−9^ substitutions per site per year according to Ossowski *et al*.^47^.

### Transcriptome sequencing of spinach germplasm collection

A total of 120 cultivated (*S. oleracea*) and wild (*S. tetrandra* and *S. turkestanica*) spinach accessions including nine that were recently reported by Xu *et al*.^11^ were collected, among which 51 were provided by the North Central Regional Plant Introduction Station (NCRPIS), Ames, Iowa, and 69 by seed companies (Supplementary Data 1). Sample collection, total RNA extraction and RNA-Seq library construction were same as described in Xu *et al*.^11^. Specifically, the entire 20-day-old seedlings were collected and stored at −80 °C till use. Total RNA was extracted using the QIAGEN RNeasy Plant Mini Kit following the manufacturer's instructions (QIAGEN, Valencia, CA, USA). The quality and quantity of RNA were assessed by electrophoresis on 1% agarose gels and by a NanoDrop 1000 spectrophotometer (Thermo Scientific, Waltham, MA, USA), respectively. The strand-specific RNA-Seq libraries were constructed for each accession following the protocol described in Zhong *et al*.^61^ and sequenced on an Illumina HiSeq 2500 platform (Illumina Inc., USA) using the single-end mode with the read length of 100 bp.

### RNA-Seq data analysis and SNP calling

Raw RNA-Seq reads were processed using Trimmomatic^29^ (v0.33; parameters ‘SLIDINGWINDOW:4:20 LEADING:3 TRAILING:3 MINLEN:40') to remove adaptor and low quality sequences. Reads longer than 40 bp were kept and then aligned to the ribosomal RNA database (https://www.arb-silva.de/) using bowtie^62^ (v1.0.0; parameter ‘-v 3') and the aligned reads were discarded. For gene expression analysis, the resulting high-quality cleaned reads were aligned to the assembled spinach genome using TopHat^51^ allowing two mismatches. Following alignments, raw counts for each spinach gene were derived and normalized to RPKM.

To identify SNPs and small indels among the 120 spinach accessions, raw RNA-Seq reads were first processed to collapse duplicated reads into unique reads and remove adaptor and low quality sequences. The resulting reads were aligned to the spinach genome using BWA^63^ (v0.7.12-r1039; parameters ‘-n 0.04 -o 1 -e 2'). Only reads uniquely mapped (having a single best match) to the genome were kept. Following mapping, SNPs and small indels were identified based on the mpileup files generated by SAMtools^64^ (v1.1). The identified SNPs and small indels were supported by at least three distinct reads and each genotype had an allele frequency >25%.

### Phylogenetic and population structure analyses

Only SNPs with minor allele frequency (MAF) >5% and missing data <10% (a total of 11,434) were used for phylogenetic and population structure analyses. A subset of 2,769 SNPs at fourfold-degenerate sites was used to construct a maximum parsimony phylogenetic tree using PAUP*^65^ (parameters ‘search=heuristic start=stepwise addseq=random swap=tbr') with 100 bootstrap replications. Two *S. tetrandra* accessions, Sp42 and Sp43, were used as the outgroup because of their larger genetic distance from the cultivars. Principal component analysis was performed using the 11,434 SNPs with the EIGENSOFT^66^ smartpca (v13050) program with the parameter ‘-k' set to 10. Population structure analysis was performed using the program STRUCTURE^23^ (v2.3.4). To determine the most likely group number, STRUCTURE was run 20 times on 1,000 randomly selected SNPs for each *K* value from 1 to 20. The optimal *K* was selected using the Evanno method^67^. After determining the Δ*K*, the subgroup membership of each accession was determined by 15,000 iterations for each *K* (*K*= 2 to 20) using the 11,434 SNPs.

*π* was used to calculate nucleotide diversities in each *Spinacia* group. SNPs with genotype information in at least 90% of the accessions were used in the calculation. A sliding window approach was used to calculate *π* in the assembled spinach genome with a window size of 10 kb and a step size of 1 kb. LD decay was calculated based on the correlation coefficient (*r*^*2*^) of alleles using Haploview^68^ (v4.2; parameters ‘-dprime -maxdistance 1000 -minMAF 0.05 -hwcutoff 0.001').

### Selective sweep and population differentiation

Whole-genome screening of selective sweeps was performed by comparing allele frequency differentiation between *S. turkestanica* and *S. oleracea* using XP-CLR^27^ (v1.0), a method based on modelling the likelihood of multilocus allele frequency differentiation between two populations. Genetic distances between SNPs were interpolated according to their physical distances in our genetic map. XP-CLR was run for each pseudochromosome or scaffold with parameters ‘-w1 0.0005 100 100 -p0 0.7'. Final estimates were tabulated in non-overlapping 10-kb windows across the genome. Adjacent windows with high XP-CLR scores (top 3%) were grouped into a single region, which was then given a score using the maximum of region-wise XP-CLR. Regions with top 1% of XP-CLR scores were considered as potential selected sweeps. We further calculated the *π* ratios between *S. turkestanica* and *S. oleracea* (*π*_w_/*π*_c_) in 10-kb sliding windows with a step size of 1 kb and identified regions with top 50% highest *π* ratios. Three selective sweeps derived from the XP-CLR analysis were not shared with those by the *π* ratio approach and thus excluded from the final results of the putative selected regions.

The population fixation index *F*_ST_ was calculated using a sliding window approach (10-kb sliding windows with a step size of 1 kb) using the R package HIERFSTAT^69^ (v0.04-22). Genomic regions with top 1% of *F*_ST_ values between *S. oleracea* and *S. turkestanica* were identified as potential differentiated regions.

### Genome-wide association study of bolting

Bolting trait of 59 spinach accessions (20–30 replications) were evaluated in the greenhouse at Shanghai Normal University in the spring of 2015. Bolting time was monitored daily and determined as the number of days from planting to the first elongation of the floral stem observed in at least half of the used plants for each accession. Correction for population stratification and genome-wide association study for the bolting trait were performed using EIGENSOFT^66^ (version 6.0.1; parameters ‘-q YES -k 10'), and using a total of 11,434 SNPs (MAF>5% and missing data <10%). SNPs with *P*<0.0001 were considered significantly associated.

### Data availability

This Whole Genome Shotgun project has been deposited at DDBJ/ENA/GenBank under the accession LZYP00000000. The version described in this paper is version LZYP01000000. Raw sequence reads of genome and transcriptome sequencing have been deposited in NCBI sequence read archive (SRA) under accession number SRP076521. The spinach genome sequence and the annotation are also available at SpinachBase (http://www.spinachbase.org). The authors declare that all other data supporting the findings of this study are available from the corresponding authors on request.

## Additional information

**How to cite this article:** Xu, C. *et al*. Draft genome of spinach and transcriptome diversity of 120 *Spinacia* accessions. *Nat. Commun.* **8,** 15275 doi: 10.1038/ncomms15275 (2017).

**Publisher's note:** Springer Nature remains neutral with regard to jurisdictional claims in published maps and institutional affiliations.

## Supplementary Material

Supplementary Information Supplementary Figures, Supplementary Tables, Supplementary Note and Supplementary References

Supplementary Data 1 Geographic information and statistics of the RNA-Seq libraries for the 120 Spinach accessions. Libraries were sequenced using the single-end mode with the read length of 100 bp.

Supplementary Data 2 List of plant genome sequences used in the comparative genomic analysis

Supplementary Data 3 Spinach disease resistance genes

Supplementary Data 4 Genes in the candidate selective sweep regions

Supplementary Data 5 Markers and QTLs overlap with or close to (<200 kb) potential selective sweeps and differentiated regions.

Supplementary Data 6 Genes in the highly differentiated regions (top 1% FST) between *S. turkestanica* and *S. oleracea*.

## Figures and Tables

**Table 1:** Summary of spinach genome assembly.

	**Contig**		**Scaffold**		**Super scaffold**	
	**Size (bp)**	**Number**	**Size (bp)**	**Number**	**Size (bp)**	**Number**
N90	1,554	71,235	5,121	6,093	11,883	3,878
N80	4,762	40,488	81,129	2,246	103,609	1,409
N70	8,537	27,590	155,870	1,489	205,174	730
N60	12,418	19,540	229,174	1,033	395,765	370
N50	16,570	13,759	319,471	711	919,290	201
N25	31,281	4,483	626,780	218	3,106,702	51
Longest	185,618	1	3,292,865	1	9,343,782	1
Total	830,856,911	215,350	869,796,885	78,264	996,306,834	77,702

Only contigs and scaffolds ≥500 bp were included in the genome assembly.
