# Supplementary material for: Draft genome of spinach and transcriptome diversity of 120 Spinacia accessions
Source: Nat Commun. 2017 May 24;8:15275. doi: 10.1038/ncomms15275 (PMC5458060; doi:10.1038/ncomms15275)
Supplement: Supplementary Information — Supplementary Figures, Supplementary Tables, Supplementary Note and Supplementary References [file ncomms15275-s1.pdf]

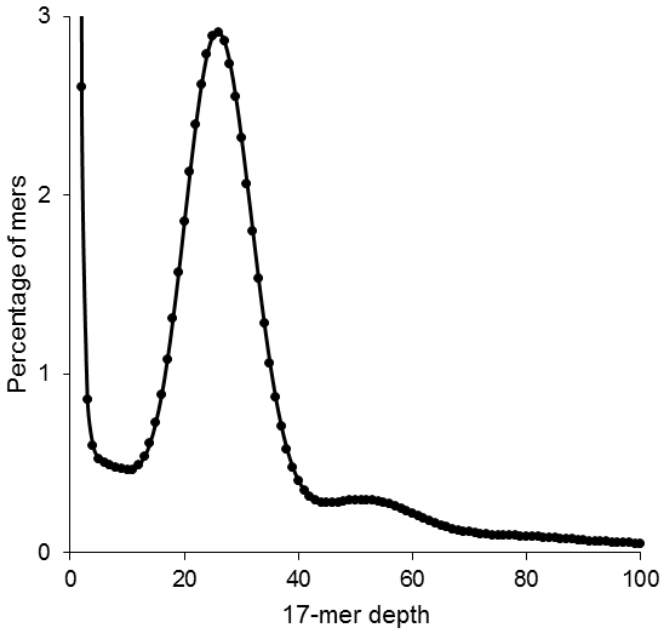

Supplementary Figure 1. 17-mer depth distribution of the Illumina reads. Illumina reads from the 1-kb insert library were used in the analysis. A total of 26,234,801,500 17-mers were obtained and the peak depth was 26. Spinach genome size was estimated to be 1,009,030,827 bp, based on the formula: Total number of K mer/Position of peak depth = 26,234,801,500 / 26 = 1,009,030,827 bp.

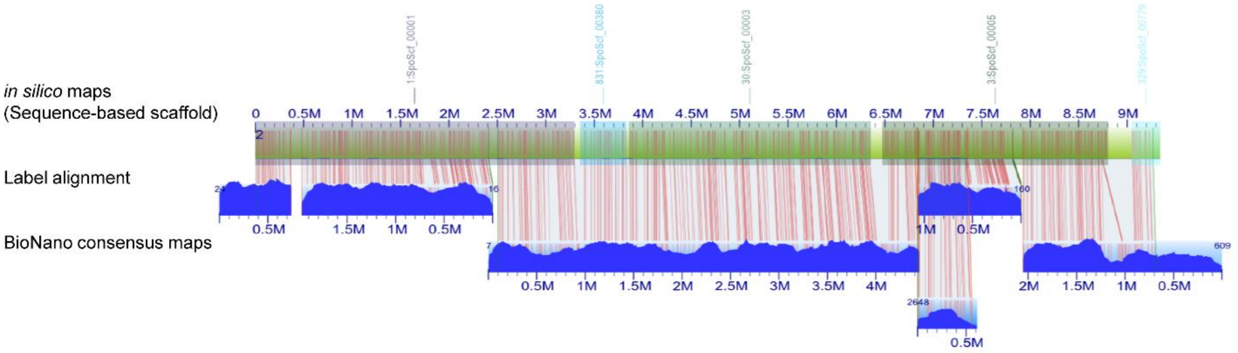

Supplementary Figure 2. Example of alignments between *in silico* maps (scaffolds) and BioNano consensus maps. This example shows the largest super-scaffold, which consists of five scaffolds (SpoScf_00001, SpoScf_00380, SpoScf_00003, SpoScf_00005, and SpoScf_00779).

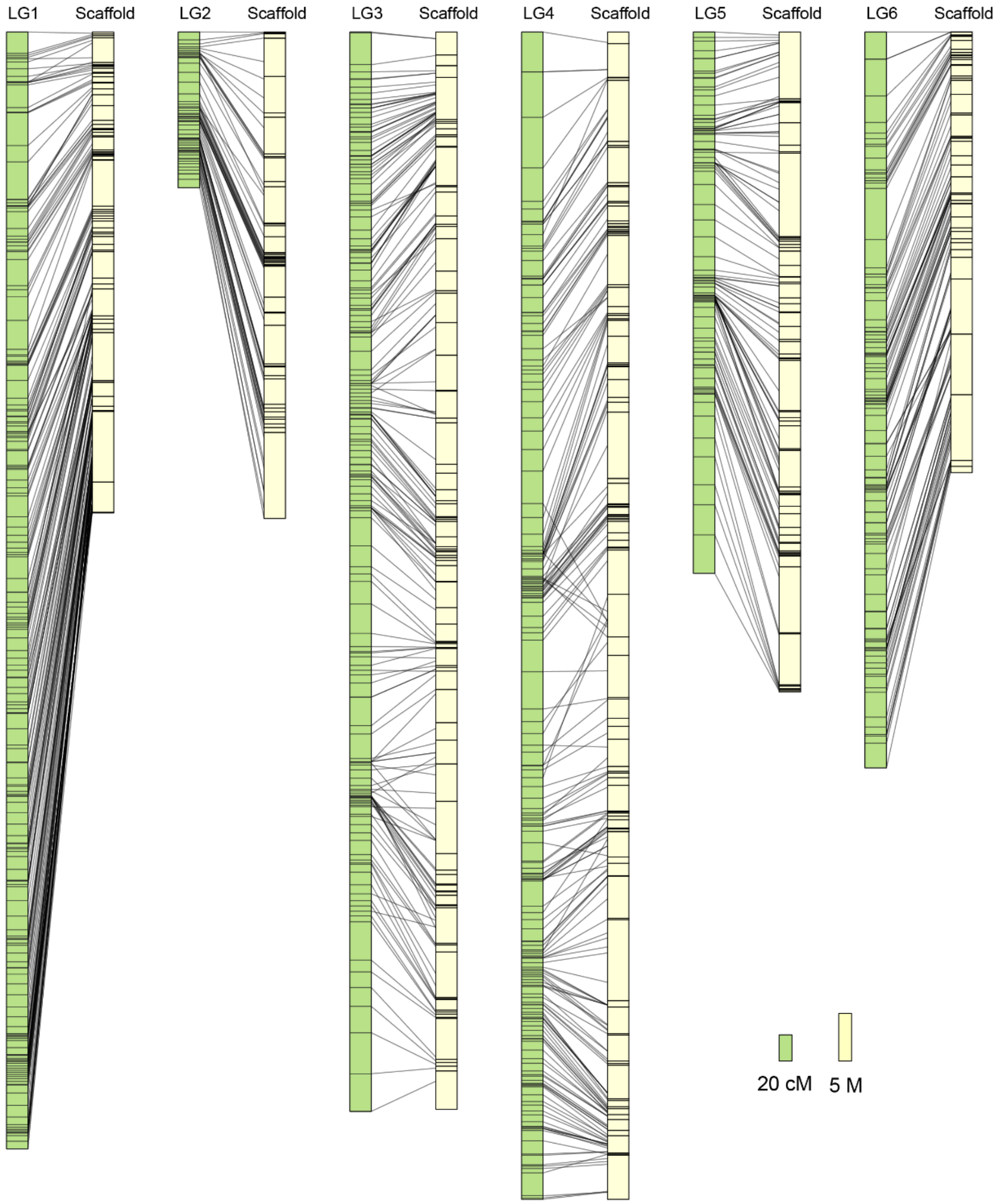

Supplementary Figure 3. Anchoring the spinach genome assembly to the reference genetic map. A total of 463 Mb spinach genome scaffolds (yellow) were anchored to the six linkage groups (green) with 870 SNP markers.

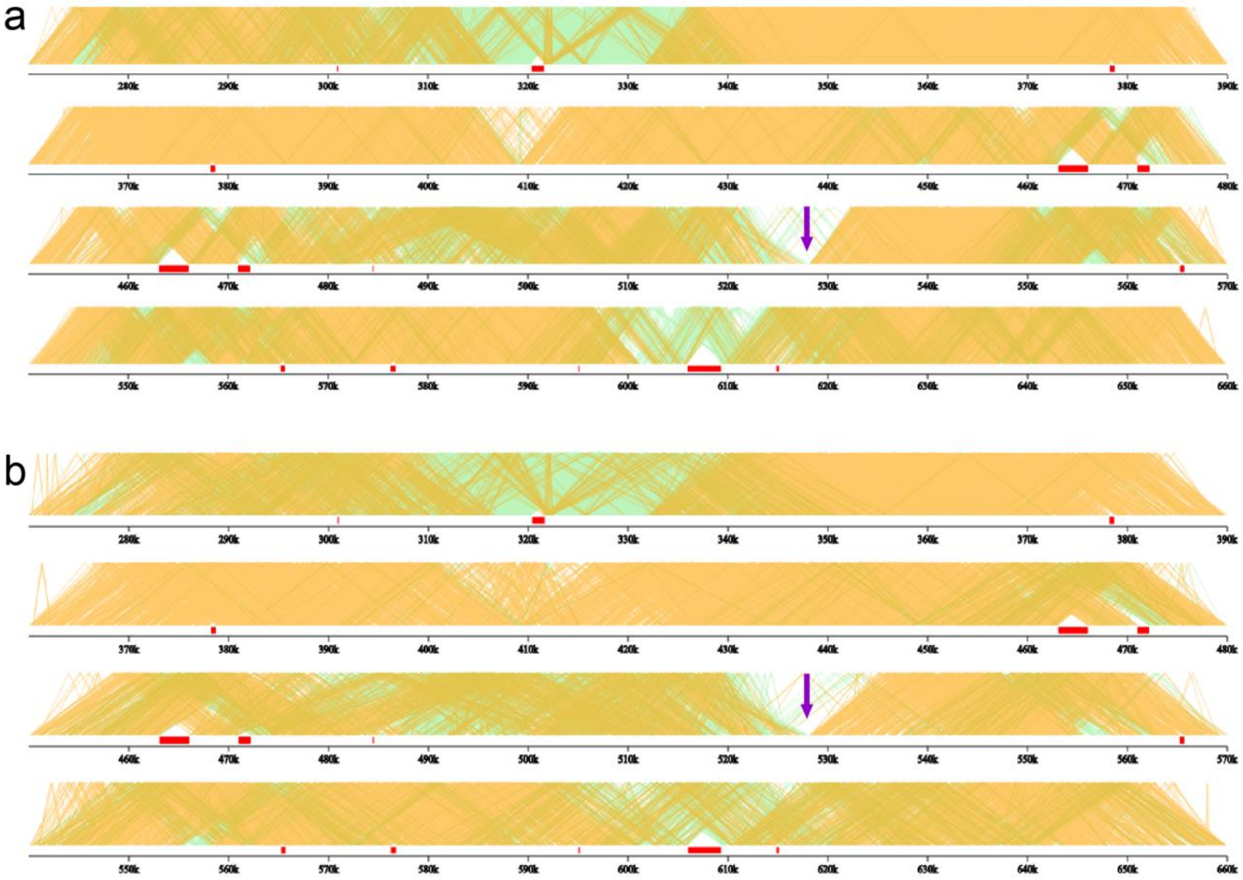

Supplementary Figure 4. Example of alignments of mate-pate reads from the 10-kb (a) and 15-kb (b) insert libraries to a scaffold (SpoScf_00179) that showed inconsistency with the linkage groups. Break point (pointed by arrows) suggested by the genetic map was supported by the alignments. Yellow lines indicate unique alignments while green lines show multiple alignments. Red bars indicated the gapped regions in the scaffold.

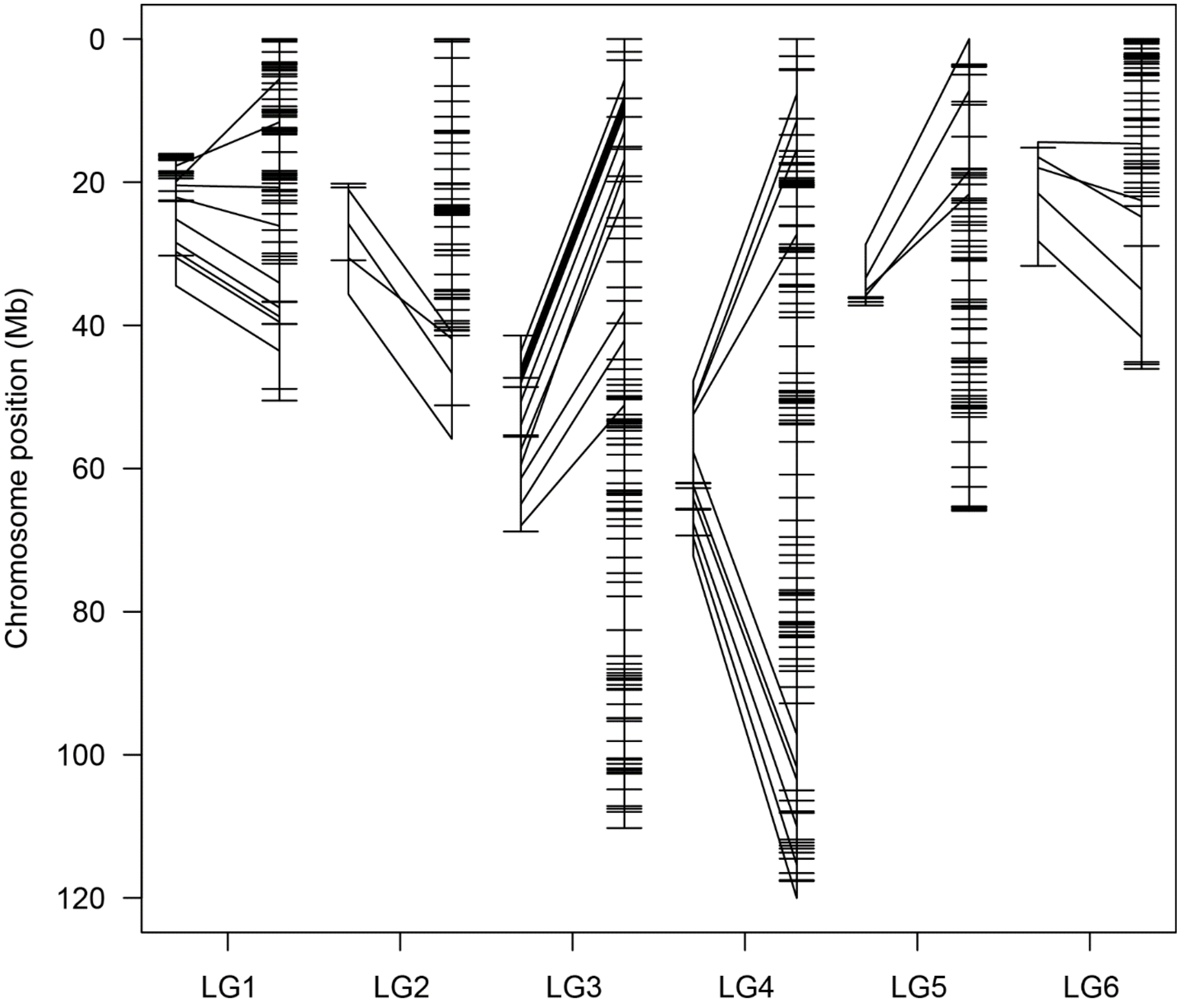

Supplementary Figure 5. Comparison of spinach genome anchoring using a published genetic map (Chan-Navarrete et al., 2016; left) and the map generated under this study (right). Horizontal bars indicate scaffolds that were anchored only to one of the two maps, and scaffolds present in both maps are linked with lines.

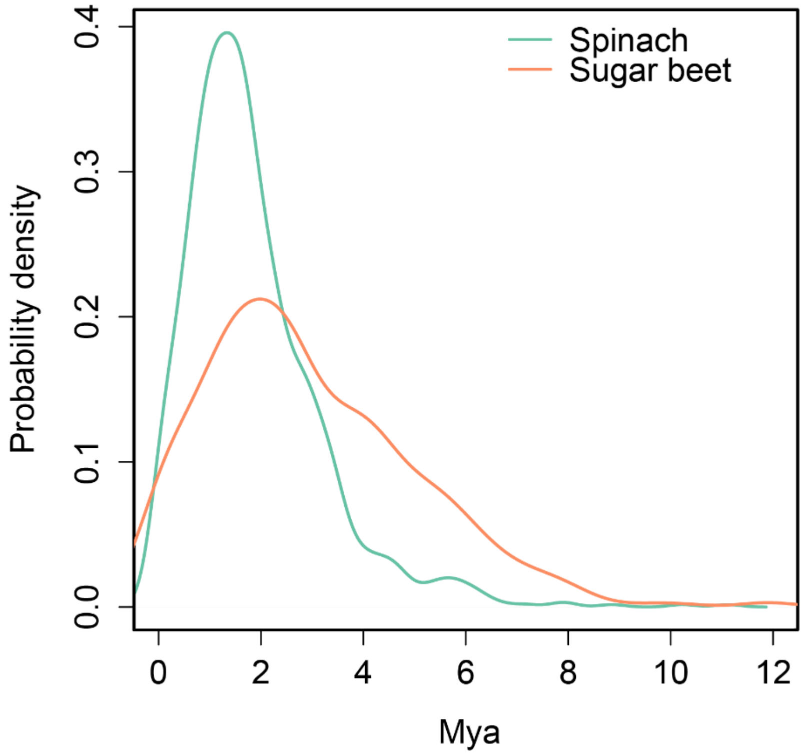

Supplementary Figure 6. Distribution of LTR insertion time of spinach and sugar beet. The probability density of LTR insertion time was estimated using the 'density' function in R. Mya: million years ago.

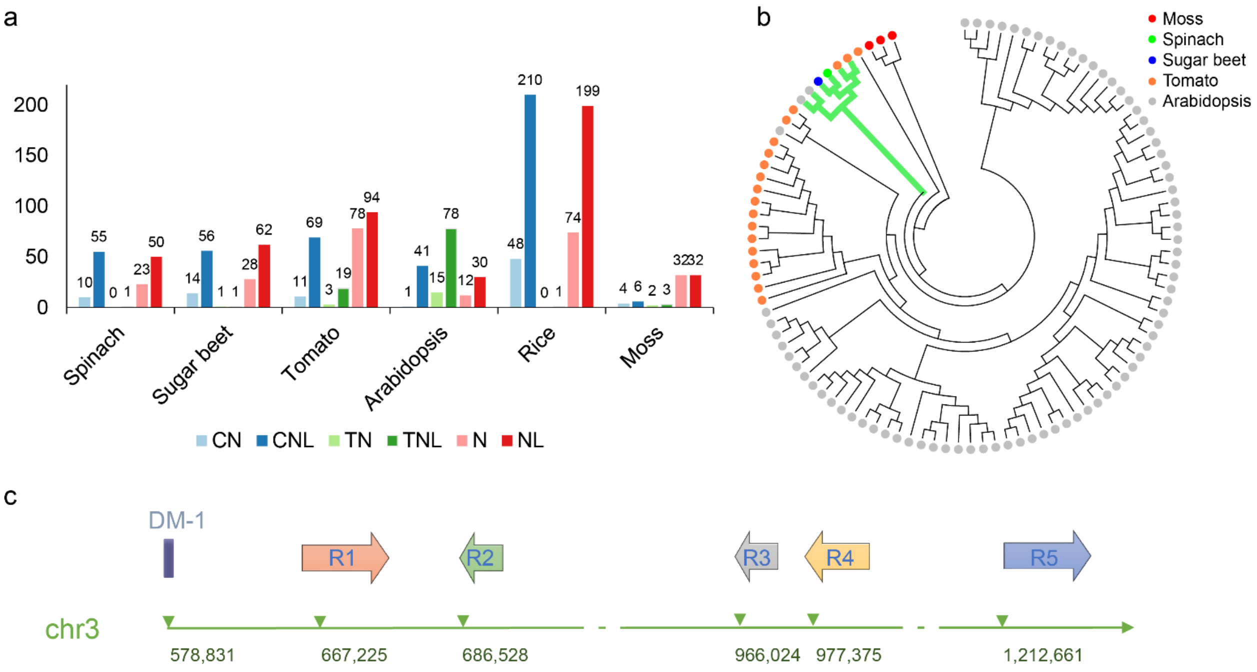

Supplementary Figure 7. NBS R genes in spinach and other five plant genomes. (a) Classification of NBS R genes in six plant genomes. CN: CC-NBS; CNL: CC-NBS-LRR; TN: TIR-NBS; TNL: TIR-NBS-LRR; N: NBS; NL: NBS-LRR. **(b)** Phylogenetic tree of TNLs. The clade highlighted in green consists of the common TNLs in four eudicots. The tree was generated using protein sequences of the conserved NB-ARC domain. **(c)** Genome region of the DM-1 marker tightly linked to downy mildew resistance. R1-R5 correspond to R genes *Spo12736*, *Spo12784*, *Spo12903*, *Spo12905* and *Spo12821*, respectively.

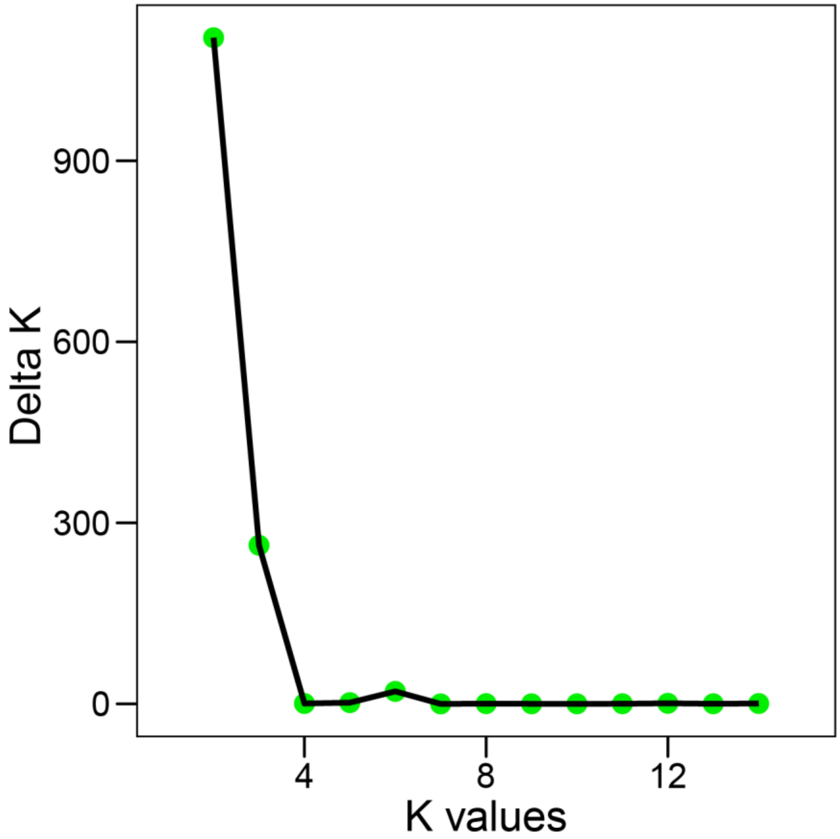

Supplementary Figure 8. ***ΔK*** **analysis for different number of clusters (*K*) for the spinach population consisting of 120 accessions.**

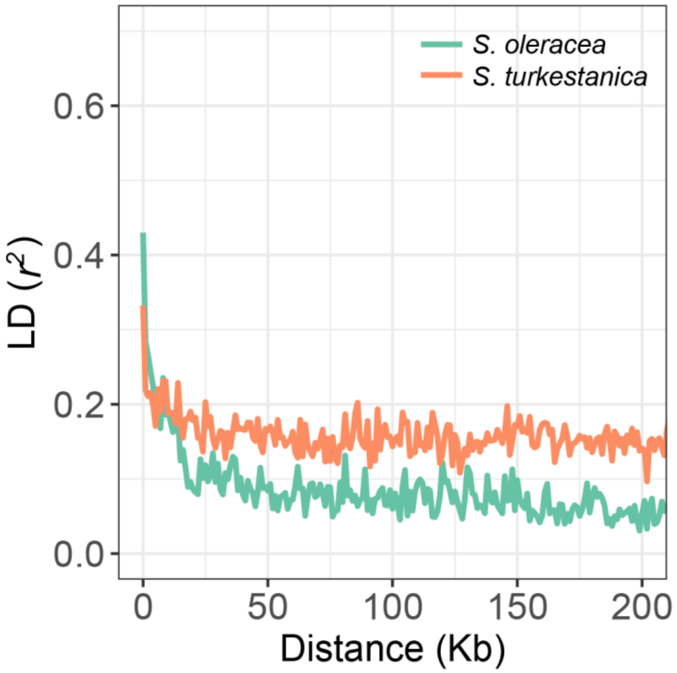

Supplementary Figure 9. LD decay determined by squared correlations of allele frequencies (r^2) against physical distance (kb) between polymorphic sites in cultivated (*S. oleracea*) and wild spinach (*S. turkestanica*).

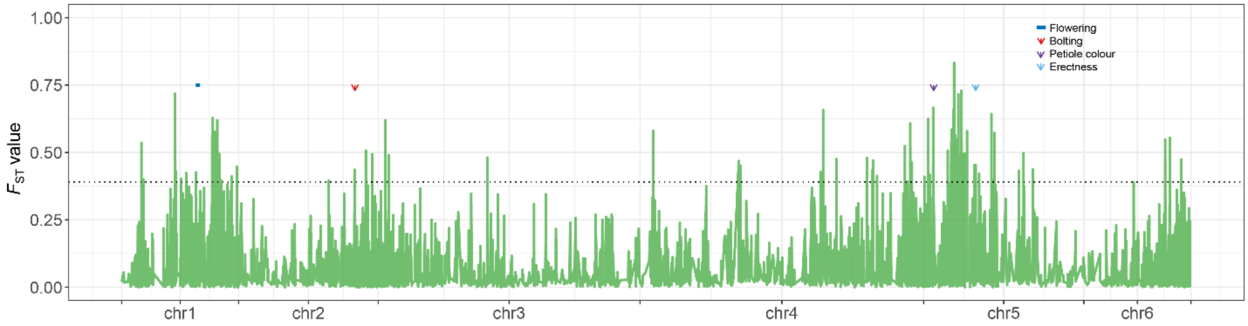

Supplementary Figure 10. Genome-wide distribution of F_{ST} values between *S. oleracea* and *S. turkestanica*. The horizontal dashed line indicates the top 1% threshold. Arrows and the short interval indicate positions of known SNP markers and QTL, respectively, for different traits.

Supplementary Table 1. Summary of spinach genome sequencing data.

Library type	Library insert size	Read length	Raw data		Cleaned data		Depth
			No. Reads	Total bases	No. Reads	Total bases	
Paired-end	150 bp	151	218,321,078	32,966,482,778	185,735,392	22,677,346,322	22.47
	200 bp	150	160,125,228	24,018,784,200	139,538,642	20,042,276,909	19.86
	300 bp	150	310,319,806	46,547,970,900	277,300,292	39,821,177,481	39.46
	500 bp	150	238,442,978	35,766,446,700	210,052,456	30,113,139,215	29.84
	1 kb	150	219,784,748	32,967,712,200	203,927,628	29,497,794,666	29.23
Mate-pair	3-5 kb	150	147,464,858	22,119,728,700	90,828,046	10,816,700,919	10.72
	8-10 kb	150	168,854,710	25,328,206,500	86,191,412	9,757,240,510	9.67
	15-20 kb	150	159,372,606	23,905,890,900	60,457,894	6,792,434,624	6.73
	**Total**		**1,622,686,012**	**243,621,222,878**	**1,254,031,762**	**169,518,110,646**	**168.00**

Supplementary Table 2. Classification of repetitive sequence identified in spinach genome.

Class		Family	Subfamily	Count	Masked (bp)	Masked (%)
TEs	Class I	LTR	Caulimovirus	25	26,527	0.00
			Copia	232,040	211,625,571	25.47
			ERV1	752	309,021	0.04
			ERV4	106	102,270	0.01
			ERVK	839	475,253	0.06
			ERVL	96	72,432	0.01
			Gypsy	226,830	228,869,104	27.55
			Ngaro	42	3,892	0.00
			Pao	2,701	1,695,276	0.20
			Others	28,777	26,941,544	3.24
		LINE	CRE-II	2,415	2,269,190	0.27
			I	446	240,244	0.03
			Jockey	4,845	1,750,041	0.21
			L1	27,422	19,131,990	2.30
			L1-Tx1	134	23,562	0.00
			R1	309	88,555	0.01
			R2	103	35,023	0.00
			RTE-BovB	1,363	291,969	0.04
			Tad1	359	104,960	0.01
		SINE	ID	47	4,621	0.00
			tRNA-Core	91	13,133	0.00
			tRNA-L2	317	43,065	0.01
			tRNA-RTE	1,217	164,799	0.02
			Others	509	181,679	0.02
	Class II	DNA	CMC-Chapaev	2,641	2,548,498	0.31
			CMC-EnSpm	40,560	15,097,821	1.82
			CMC-Transib	334	95,946	0.01
			Dada	39	6,938	0.00
			Ginger	30	6,833	0.00
			MULE-MuDR	2,505	1,661,526	0.20
			Maverick	897	719,376	0.09
			MuLE-MuDR	10,102	6,632,465	0.80
			PIF-Harbinger	2,468	944,677	0.11
			PIF-ISL2EU	48	5,811	0.00
			PiggyBac	107	33,380	0.00
			TcMar-Stowaway	47,335	9,652,860	1.16
			hAT	3,060	746,899	0.09
			hAT-Ac	19,357	8,702,711	1.05
			hAT-Tag1	6,398	1,734,632	0.21
			hAT-Tip100	2,651	867,613	0.10
			Others	2,770	754,051	0.09
		MITE		102,617	24,175,497	2.91
		Helitron		1,549	897,226	0.11
	Unknown			144,131	43,656,767	5.25
	**Total interspersed**			**921,384**	**613,405,248**	**73.83**
Satellite				1,143	313,648	0.04
Simple repeat				14,346	4,709,928	0.57
**Total**				**936,873**	**618,428,824**	**74.43**

Supplementary Table 3. Statistics of protein-coding genes in spinach and sugar beet

Category	Gene		CDS		Exon	
	Spinach	Sugar beet	Spinach	Sugar beet	Spinach	Sugar beet
Number	25,495	26,923	25,495	26,923	134,932	120,208
Mean length	5,717	5,392	1,157	1,057	219	237
Max length	181,202	142,131	16,230	16,314	8,286	7,551
Min length	108	140	96	3	1	3
Mean exon number	5	4	NA	NA	NA	NA

Supplementary Table 4. Functional annotation of spinach protein coding genes.

Databases	No. matched	%matched
GenBank nr	21,964	86.15
TrEMBL Plant	21,072	82.65
Swiss-Prot Plant	14,759	57.89
TAIR10	19,206	75.33
Interpro	19,620	76.96
Pfam	18,725	73.45
Blast2GO nr	17,744	69.60
Pathway (Metacyc)	2,717	10.66
Blast2GO nr with EC	4,333	17.00

Supplementary Table 5. Summary of transcription factors in spinach and other six plant genomes

TF families	Spinach	Sugar beet	Tomato	Grape	Watermelon	Arabidopsis	Rice
AP2/ERF-AP2	10	10	22	15	16	13	14
AP2/ERF-ERF	75	74	140	81	125	124	139
AP2/ERF-RAV	3	3	3	1	4	4	4
Alfin-like	4	4	10	6	6	7	9
B3	60	44	74	29	31	66	54
B3-ARF	14	14	22	17	15	22	27
BBR-BPC	4	4	6	5	4	7	4
BES1	6	7	9	6	6	8	6
BSD	1	1	1	1	0	2	0
C2C2-CO-like	9	7	13	6	11	16	11
C2C2-Dof	22	21	33	22	36	36	30
C2C2-GATA	20	16	30	19	22	30	25
C2C2-LSD	3	3	3	3	4	3	5
C2C2-YABBY	7	7	9	7	8	6	8
C2H2	61	76	116	76	100	106	121
C3H	50	56	62	49	46	57	57
CAMTA	3	4	6	4	4	6	6
CPP	5	5	4	6	4	8	11
CSD	3	4	5	2	3	4	2
DBB	4	4	5	6	6	3	8
DBP	3	3	3	4	2	2	4
E2F-DP	4	4	8	7	6	8	7
EIL	3	4	9	2	4	6	9
FAR1	56	21	28	19	3	17	5
GARP-ARR-B	7	6	11	10	9	9	6
GARP-G2-like	29	28	53	39	37	41	46
GRAS	27	28	54	43	37	34	60
GRF	9	8	13	8	8	9	12
GeBP	3	2	11	1	7	20	17
HB-BELL	9	9	14	12	10	13	13
HB-HD-ZIP	24	23	50	30	39	42	40
HB-KNOX	4	5	8	8	8	8	9
HB-PHD	0	2	2	2	2	2	1
HB-WOX	10	8	10	11	11	16	14
HB-other	8	8	21	10	8	11	14
HRT	1	1	1	1	1	2	1
HSF	16	17	26	19	24	24	25
LFY	1	1	1	1	1	1	1

LIM	5	4	11	6	8	6	6
LOB	30	23	47	44	36	43	36
MADS-M-type	25	16	74	18	18	69	37
MADS-MIKC	27	27	31	36	21	39	35
MYB	77	67	140	139	111	142	117
MYB-related	45	40	76	53	50	58	65
NAC	59	52	96	71	79	111	135
NF-X1	2	2	2	3	2	2	2
NF-YA	6	6	10	7	7	10	11
NF-YB	10	10	27	15	10	13	13
NF-YC	5	8	16	5	7	14	16
NOZZLE	0	0	0	1	1	1	0
OFP	15	12	24	8	17	17	31
PLATZ	8	8	21	13	10	12	15
RWP-RK	9	7	10	8	8	14	12
S1Fa-like	1	1	1	2	2	3	2
SAP	1	1	2	1	1	1	0
SBP	20	12	17	19	16	17	19
SRS	5	4	8	5	9	10	5
STAT	1	1	1	1	1	2	1
TCP	17	15	36	15	27	24	20
TUB	5	5	11	13	9	11	15
Tify	7	6	17	15	11	15	17
Trihelix	23	22	25	23	26	26	26
ULT	0	0	3	1	2	2	2
VOZ	2	2	2	2	2	2	2
WRKY	53	45	81	59	57	73	94
Whirly	2	2	2	2	2	3	2
bHLH	102	92	143	102	108	137	135
bZIP	55	46	66	45	59	71	90
zf-HD	7	12	22	10	14	17	14
**Total**	**1202**	**1090**	**1918**	**1330**	**1399**	**1758**	**1800**

Supplementary Table 6. Classification of receptor like kinases (RLKs) in spinach and other six plant genomes.

RLKs	Spinach	Sugar beet	Tomato	Grape	Watermelon	Arabidopsis	Rice
C-LEC	1	1	1	1	1	1	1
CR4L	7	7	9	7	7	7	17
CrRLK1L-1	15	14	28	10	16	15	18
DLSV	131	22	116	186	58	91	147
DSLV	0	0	0	0	0	1	0
Extensin	7	7	6	6	6	5	6
L-LEC	20	24	27	29	25	45	101
LRK10L-2	8	5	15	11	4	13	44
LRR-I-1	13	8	8	22	19	48	39
LRR-I-2	2	2	2	3	3	2	2
LRR-II	10	10	13	14	12	14	12
LRR-III	33	29	44	34	36	47	45
LRR-IV	3	3	2	2	3	3	3
LRR-IX	5	6	8	6	6	5	3
LRR-V	7	6	9	8	8	9	12
LRR-VI-1	4	4	6	5	5	5	4
LRR-VI-2	5	5	6	5	5	8	10
LRR-VII-1	4	4	5	4	5	5	8
LRR-VII-2	1	1	2	2	2	3	3
LRR-VII-3	2	2	3	2	1	2	1
LRR-VIII-1	8	11	11	5	5	8	9
LRR-XI-1	37	37	53	81	40	34	49
LRR-XI-2	2	2	3	1	2	2	2
LRR-XII-1	22	18	55	34	19	8	100
LRR-XIIIa	3	3	3	4	6	4	3
LRR-XIIIb	1	1	2	2	2	3	3
LRR-XIV	1	1	2	2	3	2	3
LRR-XV	2	2	2	1	2	2	2
LRR-Xa	4	4	6	5	2	4	3
LRR-Xb-1	10	6	10	8	7	9	24
LRR-Xb-2	1	1	2	1	1	1	1
LysM	15	15	16	12	11	6	12
PERK-1	15	13	14	8	7	15	11
PERK-2	3	3	6	8	4	3	3
RKF3	6	3	2	3	2	2	3
RLCK-II	1	2	2	1	2	1	1
RLCK-IV	4	3	3	3	3	3	6
RLCK-IXa	1	1	2	4	1	2	3
RLCK-IXb	14	18	16	11	13	20	26
RLCK-Os	1	2	0	4	2	0	8
RLCK-V	9	8	8	8	9	11	14
RLCK-VI	11	10	15	14	13	14	10
RLCK-VIII	7	5	7	4	5	11	10
RLCK-VIIa-1	12	13	19	13	12	15	15
RLCK-VIIa-2	32	20	37	26	32	33	38

RLCK-VIIb	1	1	0	1	1	1	1
RLCK-X	3	1	2	3	2	4	3
RLCK-XI	3	3	3	3	3	4	3
RLCK-XII-1	6	6	7	7	8	13	5
RLCK-XII-2	1	9	2	5	0	13	0
RLCK-XIII	1	1	2	1	1	2	5
RLCK-XV	1	1	3	2	4	2	4
RLCK-XVI	1	1	1	1	1	1	1
RLCK-XVII	0	0	0	0	0	2	0
SD-2b	29	34	39	34	23	10	105
Singleton	1	1	1	1	1	1	0
URK-1	2	2	3	2	2	3	3
URK-2	0	0	3	3	1	0	3
URK-3	1	0	1	1	0	1	1
WAK	44	51	14	19	8	21	122
WAK_LRK10L-1	12	13	19	10	13	9	8

Supplementary Table 7. Summary of transcriptome SNPs and small indels in spinach accessions and different sub-groups.

Group	Sample	Sample size	Genotype percentage	No. small indels	No. SNPs	Total
**Total**	all three species	120	All[a]	12,618	420,545	433,163
			50%[b]	6,229	274,399	280,628
			90%[c]	3,048	142,941	145,989
**Sub1**	*S. oleracea*	107	All[a]	6,333	192,515	198,848
			50%[b]	2,339	115,401	117,740
			90%[c]	829	50,872	51,701
**Sub2**	*S. tetrandra* (excluding Sp39 and Sp40)	3	All[a]	2,652	117,299	119,951
			90%[c]	1,618	88,027	89,645
**Sub3**	*S. turkestanica* (excluding Sp47 and Sp48)	6	All[a]	1,958	51,977	53,935
			50%[b]	1,543	44,132	45,675
			90%[c]	419	16,081	16,500
**Sub2_1**	*S. tetrandra*	5	All[a]	5,167	200,401	205,568
			50%[b]	4,168	171,621	175,789
			90%[c]	1,608	86,693	88,301
**Sub3_1**	*S. turkestanica*	8	All[a]	2,323	64,347	66,670
			50%[b]	1,631	50,648	52,279
			90%[c]	408	17,199	17,607

[a]All SNPs without filtering based on missing genotype rate
[b]Excluding SNPs and small indels with more than 50% missing genotype rate
[c]Excluding SNPs and small indels with more than 10% missing genotype rate

Supplementary Table 8. Summary of potential effects of SNPs and small indels.

	No. SNPs	No. Genes
Upstream (2 kb upstream of translational start site)	33,020	8,306
Downstream (2 kb downstream of translational stop site)	46,133	9,994
Intergenic	23,060	0
Splice site acceptor or donor	5,524	3,099
Intronic	39,420	7,909
Synonymous	154,349	15,236
Non-synonymous	117,029	15,682
Stop codon <==> Non-stop codon	1,669	1,515
Start codon => Non-start codon	133	120
Stop codon change[a]	145	145
Start codon change[b]	51	51
Undetermined[c]	12	6
Total	420,545	18,540
	**No. small indels**	**No. Genes**
Upstream (2 kb upstream of translational start site)	2,715	1,991
Downstream (2 kb downstream of translational stop site)	3,649	2,602
Intergenic	1,025	0
Intronic	2,792	1,946
Frame shift or indel of an amino acid	2,437	1,358
Total	12,618	6,389

[a] A stop codon changes to another stop codon
[b] A start codon changes to another start codon
[c] Undetermined base in the reference genome sequence

Supplementary Table 9. Genetic diversity in different spinach populations

Group Description	Species	No. accessions	π per kb
All cultivars	*S. oleracea*	107	0.6687
All wild	*S. turkestanica & S. tetrandra*	13	4.1554
Wild accessions (excludingSp47 & Sp48)	*S. turkestanica & S. tetrandra*	11	4.6847
Wild *S. turkestanica* accessions	*S. turkestanica*	8	0.8217
Wild *S. turkestanica* accessions (excluding Sp47 & Sp48)	*S. turkestanica*	6	0.8323
Wild *S. tetrandra accessions*	*S. tetrandra*	5	7.2573
Wild *S. tetrandra* accessions (excluding Sp39 & Sp40)	*S. tetrandra*	3	6.4027

Supplementary Note 1. Genetic map construction and genome anchoring

An F2 mapping population, consisting of 109 individuals, were generated by crossing a pair of F1 siblings, which were derived from a cross between Sp75 (male), the line used to generated the reference genome sequence, and a gynoecious line Sp73 (female). All the plant materials were grown in the greenhouse of Shanghai Normal University in the spring of 2015. Genomic DNA was extracted from young healthy leaves of F2 individuals and the parents using the cetyltrimethylammonium bromide (CTAB) method. DNA concentration was measured using an ND-2000 spectrophotometer (NanoDrop, Wilmington, DE, USA) and quality was assessed by electrophoresis using 1% agarose gels with a lambda DNA standard. Genotyping of these plants was performed following the genotyping-by-sequencing (GBS) protocol[1], using ApeKI as the restriction enzyme. The resulting libraries were multiplexed and sequenced on a HiSeq 2500 system (Illumina Inc. USA) with single-end mode and read length of 100 bp. The GBS reads were mapped to the spinach reference genome sequence using bwa aln[2] (v0.7.12) with default parameters. We compared and used three different programs, GATK[3] (v3.6-0-g89b7209), TASSEL-GBS (v2) and TASSEL-GBS[4] (v1), to identify SNPs from the GBS data. The minimum genotype quality was set to 60 for both TASSEL pipelines and 30 for the GATK pipeline. The resulting SNPs from the three programs were integrated and concatenated, and a total of 21,792 biallelic SNPs were obtained. The segregation ratio tests (1:2:1) were performed on these SNPs using the Chi-square analysis (P <0.001), resulting in the removal of 86% of the biallelic SNPs. SNP sites with missing genotype rate greater than 0.3 were excluded from the analysis and adjacent SNP sites (distance smaller than 1 Mb) with uniform genotype patterns across the population were grouped as one SNP marker. A total of 870 markers were used to construct a linkage map using the minimum spanning tree (MST) algorithm[5] implemented in the R package ASMap (https://cran.r-project.org/web/packages/ASMap/).

The resulting genetic map consisted of six linkage groups (LGs), corresponding to the six spinach chromosomes. The LGs had an estimated total genetic length of 3679.4 cM and an average of approximately 4.23 cM per marker (**Supplementary Fig. 3**).

Using the newly generated genetic map, we were able to anchor 439 scaffolds to the six spinach LGs, covering 463.4 Mb (47%) of the 996 Mb assembled genome and more than 60% of the total gene space (**Supplementary Fig. 3**). We manually checked the assembled scaffolds that were not consistent with LGs, by examining the alignments of the mate-pair reads from two

large-insert libraries (10 kb and 15 kb). Six scaffolds with conflicting marker positions on LGs were broken based on the evidence from the alignments **(Supplementary Fig. 4)**. Several other inconsistencies between genetic map and the genome scaffolds could still be observed (**Supplementary Fig. 3**), which could be mainly due to the errors introduced during genetic map construction.

Furthermore, we compared a previously published spinach genetic map[6], which consists of 283 SNP makers, to our genome assembly. A total of 279 markers could be uniquely mapped to the assembly, which anchored 87 scaffolds of a total length of 130.3 Mb (13% of the assembled genome), among which 123.8 Mb (95% of 130.3 Mb) were largely consistent with our anchoring results **(Supplementary Fig. 5**), supporting the high accuracy of our genome anchoring. Since only a very small portion of scaffolds (6.5 Mb) that were anchored by the genetic map of Chan-Navarrete et al.[6] were not covered by our pseudochromosomes, no further effort was made to integrate the 6.5-Mb scaffolds into our final pseudochromosomes.

The rate (47%) of assembled spinach scaffolds that could be anchored to the genetic map is relatively low. This could be due mainly to the following two reasons: 1) the genetic diversity between the two parents, both of which are cultivated species, is very low. This is the same for the genetic map of Chan-Navarrete et al[6]; and 2) spinach is a naturally dioecious species, therefore the F2 population in our mapping population had to be generated by crossing two F1 siblings with different sex types. This could be the main reason that a large portion of the SNP markers (86%) that did not pass the segregation ratio test (1:2:1; $p < 0.001$). Mapping populations derived from parents that are distantly related, e.g., one cultivated and one wild species, would greatly help to generate high-density genetic maps for spinach. However, currently very limited number of accessions are available for the two wild relatives of spinach, *Spinacia turkestanica* and *S. tetrandra*. In addition, progenies from crosses of cultivated and wild (especially *S. tetrandra*) spinaches often show highly reduced pollen fertility[7], make it difficult to develop mapping populations with high genetic diversity.

Supplementary References

1. Elshire, R. J., *et al.* A robust, simple genotyping-by-sequencing (GBS) approach for high diversity species. *PLoS One* **6**, e19379 (2011).
2. Li, H. & Durbin, R. Fast and accurate short read alignment with Burrows-Wheeler transform. *Bioinformatics* **25**, 1754-1760 (2009).
3. McKenna, A., *et al.* The Genome Analysis Toolkit: a MapReduce framework for analyzing next-generation DNA sequencing data. *Genome Res.* **20**, 1297-1303 (2010).
4. Glaubitz, J. C., *et al.* TASSEL-GBS: a high capacity genotyping by sequencing analysis pipeline. *PLoS One* **9**, e90346 (2014).
5. Wu, Y., Bhat, P., Close, T. J. & Lonardi, S. Efficient and accurate construction of genetic linkage maps from minimum spanning tree of a graph. *PLoS Gen.* **4**, 1-11 (2008).
6. Chan-Navarrete, R., Dolstra, O., van Kaauwen, M., Lammerts van Bueren, E. T. & van der Linden, C. G. Genetic map construction and QTL analysis of nitrogen use efficiency in spinach (*Spinacia oleracea* L.). *Euphytica* **208**, 621-636 (2015).
7. Fujito, S., Takahata, S., Suzuki, R., Hoshino, Y., Ohmido, N. & Onodera, Y. Evidence for a common origin of homomorphic and heteromorphic sex chromosomes in distinct *Spinacia* species. *G3* **5**, 1663-1673 (2015).
